# Protective effects of Zhuyeqing liquor on the immune function of normal and immunosuppressed mice in vivo

**DOI:** 10.1186/1472-6882-13-252

**Published:** 2013-10-03

**Authors:** Hong-ying Gao, Guo-yu Li, Jian Huang, Ying Han, Fu-zhou Sun, Xiao-wei Du, Li-juan An, Hang-yu Wang, Jin-hui Wang

**Affiliations:** 1 School of Traditional Chinese Materia Medica, Shenyang Pharmaceutical University, Shenyang 110016, P.R. China; 2 School of Pharmacy, Shihezi University, Shihezi 832002, P.R. China; 3 Shanxi Xinghuacun Fen Jiu Group Co. Ltd, Shanxi 450000, P.R. China

**Keywords:** Zhuyeqing liquor, Immune function, Antioxidant effects, Cyclophosphamide

## Abstract

**Background:**

Zhuyeqing Liquor (ZYQL), a well-known Chinese traditional health liquor, has various biological properties, including anti-oxidant, anti-inflammatory, immunoenhancement and cardiovascular protective effects.

**Methods:**

The protective effects of Zhuyeqing Liquor (ZYQL) on the immune function was investigated in vivo in normal healthy mice and immunosuppressed mice treated with Cyclophosphamide (Cy, 100 mg/kg) by intraperitoneal injection on days 4, 8 and 12. ZYQL (100, 200 and 400 mg/kg) was administered via gavage daily for 14 days. The phagocytotic function of mononuclear phagocytic system was detected with carbon clearance methods, the levels of interleukin-6 (IL-6) and interferon-gamma (IFN-γ) in serum were detected with Enzyme linked immunosorbent assay (ELISA). Immune organs were weighed and organ indexes (organ weight/body weight) of thymus and spleen were calculated. Meanwhile, the activity of lysozyme (LSZ) in serum and the activity of superoxide dismutase (SOD), glutathione peroxidase (GSH-Px), catalase (CAT) in spleen tissue were measured.

**Results:**

ZYQL significantly upgrades the K value for clearance of carbon particles in normal mice treated with ZYQL (400 mg/kg) and immunosuppressed mice treated with ZYQL (100, 200 and 400 mg/kg) together with Cy (100 mg/kg) in vivo. The treatment of ZYQL (100, 200 and 400 mg/kg) effectively increased the activity of serum lysozyme as well as promoted the serum levels of IL-6 and IFN-γ in normal mice and immunosuppressed mice. Furthermore, ZYQL (100, 200 and 400 mg/kg) had an antioxidant effects in immune system by enhancing the antioxidant enzyme activity of SOD, CAT and GSH-Px in vivo. In addition, ZYQL (100, 200 and 400 mg/kg) effectively elevated the Cy-induced decreased organ index (thymus and spleen).

**Conclusions:**

The present work shows that the dose-dependent administration of ZYQL is capable of influencing immune responses, which implying that its valuable functional health may be attributed partly to its protective effects for the immune function.

## Background

Zhuyeqing Liquor (ZYQL), a well-known Chinese traditional health liquor, has been formally authorized as a functional health liquor in 1998 by Ministry of Public Health in China. It is sold commercially and is a common ingredient in a range of healthcare products. The long time history use of ZYQL has been proved that drinking ZYQL could keep body health and anti-fatigue. And with the improvement of the technology, especially with the applications of modern analytical tools and test equipment, its special composition as well as nutrition and health functions were further recognized. Through scientific experimental verification, its effectiveness of health care towards stomach, spleen and liver etc., is gradually disclosed and recognized by consumers [1].

Health food constituents possess not only nutritional value but can also have physiological effects by modulating the immune, nervous and endocrine systems. At present, substances for enhancing host defense responses were isolated from microorganisms, fungi and plants [2-4]. And some herbal medicines have been frequently used as tonic medicines to add into some special functional food in order to treat or protect from several diseases such as autoimmune diseases, inflammations and aller-gies, which are difficult to Western medicines. Moreover, plant-derived polysaccharides are the best known and most potent immunomodulatory substances and have been shown to be clinically therapeutic [5]. And their chemical properties and biological activities have been studied extensively. In the present study, ZYQL consists of twelve crude drugs: *Lophatherum gracile* Brongn. (Gramineae), *Gardenia jasminoides* Ellis (Rubiaceae), *Lysimachia capillipes* Hemsl. (Primulaceae), *Angelica sinensis* (Oliv.) Diels (Umbelliferae), *Kaempferia galanga* L. (Zingiberaceae), *Citrus reticulata* Blanco (Rutaceae), *Chrysanthemum morifolium* Ramat. (Compositae), *Amomum villosum* Lour. (Zingiberaceae), *Santalum album* L. (Santalaceae), *Eugenia caryophyllata* Thunb. (Myrtaceae), *Aucklandia lappa* Decne. (Compositae), *Lysimachia foenum-graecum* Hance (Primulaceae). These twelve crude drugs are famous Chinese herbal medicine used for the treatment of various diseases as well as a tonic medicine for thousands of years. Among them the chemical properties and biological activities of *Lophatherum gracile* Brongn., *Angelica sinensis* (Oliv.) Diels, *Gardenia jasminoides* Ellis, *Amomum villosum* Lour., *Citrus reticulata* Blanco and *Eugenia caryophyllata* Thunb. have been widely studied [6]. And the polysaccharide fractions obtained from these species have been shown to exhibit immunity regulation, anti-inflammatory, anti-hypoglycemic, anti-bacterial, anti-tumor, and anti-complementary activities [7-10]. Based on the above, we designed the method to evaluate whether ZYQL has the protective effect on immune system.

Cyclophosphamide (Cy), a multifunctional alkylating agent, is primarily used as an anticancer chemotherapeutic drug in childhood and adult malignancies. Beside a cytotoxic drug, it also suppresses the immune system and is referred to as well known immunosuppressive in case of mammals and birds [11-17]. Studies in mammals suggest that Cy affects the innate immune system by causing acute damage to the blood forming tissues in bone marrow thereby causing transient reduction in circulating PMNs (polymorphonuclear neutrophils) [15]. It also causes reduction in microsomal enzyme activity, antioxidant defenses, specific immunity through direct depletion of lymphoid tissues thus preventing the host to raise an adequate specific immune response [15,18]. Its modulation in immune reactivity is well known in mammals and the drug is regarded as a flexible means to manipulate host responsiveness to malignancies and infections in a variety of ways [11,13,19,20]. At present, Cy-induced immunosuppressed model is considered as a well characterized model, which has been extensively performed in the experiment research.

In the present work, we examined the protective effects of orally administered processed ZYQL for the immune function. Normal and immunosuppressed mice induced by Cy (100 mg/kg) were investageted in order to discover the protective effects of ZYQL on non-specific and specific immune system. To the best of our knowledge, there is no any previous studies on the protective effects of ZYQL for immune function in vivo, and this is the first report demonstrating the in vivo protective effects of orally administered ZYQL in normal healthy mice and immunosuppressed mice treated with Cy by intraperitoneal injection.

## Methods

### Drug material

The powder of ZYQL (amber powder) was provided by Shanxi XinghuaCun Fen Jiu Group Co., Ltd. (Shanxi Province, China), which was obtained from ZYQL, condensed at 50°C by vacuum rotary evaporation. The voucher specimen was deposited at Shihezi University (Xinjiang, China) and registered under the number ZYQL 2011050102. This powder was dissolved in sterilized distilled water before oral administration to the experimental animals. All doses given are the gram-weight of the administered ZYQL powder in sterilized distilled water.

### Experimental animal and design

Male inbred BALB/c mice (18±2 g) were purchased from the Experimental animal center at Xinjiang Medical University, China. Guidelines for the care and use of animals were followed and approved by the Ethical Committee of Shihezi University (Xinjiang, China). The mice were housed in specific standard laboratory conditions for one week. The conditions were kept in a temperature-controlled environment (24±1°C), a relative humidity (50±5%), and with a regular 12 h light/12 h dark cycle. All animals were fed with a standard rodent chow diet and water *ad libitum*.

Eighty BALB/c mice were randomly divided into eight groups (n = 10 in each group). Group A served as normal control and was orally given pure water for fourteen days, wherein the days 4, 8, 12 intraperitoneally injected with 10 ml/kg body weight isotonic 0.9% NaCl. Group B served as immunosuppressed control group and was orally given pure water for fourteen days, wherein the days 4, 8, 12 intraperitoneally intoxicated with 100 mg/kg Cy. Cy (Endoxan®) was purchased from the affiliated hospital of Shihezi University, Xinjiang, China. Group C, D, E were treated with the quantum satis (q.s.) dosages of ZYQL extract (100, 200 and 400 mg/kg, respectively) for fourteen days, and then wherein the days 4, 8, 12 intraperitoneally injected with 10 ml/kg body weight isotonic 0.9% NaCl. Group F, G, H were treated with the quantum satis (q.s.) dosages of ZYQL extract (100, 200 and 400 mg/kg, respectively) for fourteen days, and wherein the days 4, 8, 12 intraperitoneally intoxicated with 100 mg/kg Cy.

On the fifteenth days, the mice were bled, the blood was collected and centrifuged at 3,000 rpm for 10 min at 4°C, then stored at −80°C until further analyzed. Mice thymus, spleen and liver were immediately removed and washed with ice-cold saline, then weighed and stored at −80°C. The blood and spleen samples were assessed for their biochemical and antioxidant activities.

Other eighty BALB/c mice were used to detect the phagocytotic function of mononuclear phagocytic system with carbon clear up methods, and the experimental design was the same as the above described.

### Biochemical analysis

Serum lysozyme (LSZ), interleukin-6 (IL-6) and interferon-γ (IFN-γ) levels were measured by ELISA kits obtained from Sigma-Aldrich Chemicals Co., USA. According to the manufacturer’s protocol, the observation absorbance of the reaction was read at 450 nm.

Commercial kits used for determining glutathione peroxidase (GSH-Px), superoxide dismutase (SOD) and catalase (CAT) were obtained from Jiancheng Institute of Biotechnology (Nanjing, China).

SOD activity in spleen homogenate was assayed as described by Beauchamp and Fridovich [21] by measuring its ability to inhibit the photochemical reduction of nitro blue tetrazolium (NBT) at 560 nm. Data are expressed as SOD U/mg protein as compared with the standard.

The measurement of GSH-Px was conducted by the method of Rotruck [22], which is based on the reaction between glutathione remaining after the action of GSH-Px and 5,5′-dithiobis-(2-nitrobenzoic acid) to give a compound that absorbs light at 412 nm and the enzyme activity was calculated as μmol/g protein.

CAT activity was determined according to the method of Aebi [23]. The reaction time interval of the absorbance was monitored at 240 nm for 1 minute to measure CAT activity and the data was expressed as U/mg protein.

### Carbon clearance methods

On the fifteenth days, all the mice used to detect the phagocytotic function of mononuclear phagocytic system with carbon clear up methods were weighed, and then injected ink diluents (1:5 dilution, 0.1 ml/10 g) in the caudal vein, in the same time start to count time. Ink staining was purchased from Beijing Xizhong chemical plant (Beijing, China). Thirty μl blood was collected in the time of 2 min (T_1_) and 10 min (T_2_) from the venous plexus of the orbital cavity, respectively. Then put the blood into a sample tube, which contains 3 ml 0.1% NaCO_3_. Finally, the OD at 675 nm were determined by a spectrophotometric microplate reader. The mice were sacrificed by dislocation cervical vertebra, mice liver and spleen were obtained and weighed. The carbon clearance index A was calculated by the following equation (The manual of health food inspection and evaluation technological specification, 2003 edition):

$$\begin{array}{l} A=\text{Body}\;\text{weight}/\left(\text{Liver}+\text{Spleen}\right)\times K^{1/3}\\ K=\left(\text{logO}D_{l}-\text{logO}D_{2}\right)/\left(T_{2}-T_{l}\right)\\ OD_{1}:\text{absorbance}\;\text{in}\;2\;\text{min}\\ OD_{2}:\text{absorbance}\;\text{in}\;10\;\text{min}\\ T_{1}:\text{time}\;\text{of}\;2\;\text{min}\\ T_{2}:\text{time}\;\text{of}\;10\;\text{min} \end{array}$$

### Statistical analysis

All data were expressed as Means ± Standard Deviations, and analyzed with one-way analysis of variance (ANOVA) followed by LSD’s test and two-way ANOVA followed by LSD’s test. Statistical significance was analyzed by SPSS 13.0 software.

## Results

### Effect of ZYQL on organ coefficient in normal and immunosuppressed mice

Organ coefficients including liver, spleen and thymus coefficients were evaluated in mice. Spleen and thymus coefficients were significantly decreased in mice with Cy-treatment (*p*<0.05 and *p*<0.01, respectively, Table 1) compared with the normal group. In contrast, there is no difference in liver coefficient among all treated groups. As demonstrated in Table 1, the decrease of thymus coefficient caused by Cy treatment in mice was alleviated by ZYQL (100, 200 and 400 mg/kg) (*p*<0.01, Table 1). The protective effect against the decrease of spleen coefficient induced by Cy was observed in mice with ZYQL (200 and 400 mg/kg) (*p*<0.05, Table 1). Dose-effect correlation was observed in ZYQL groups regarding to the decrease of spleen and thymus coefficient by Cy-treatment. ZYQL did not enhance the organ coefficient in the normal mice treated with 100, 200 and 400 mg/kg of ZYQL (Table 1).

**Table 1:** Effect of ZYQL on organ coefficient in normal and immunosuppressed mice

**Treatment**	**Liver coefficient%**	**Spleen coefficient%**	**Thymus coefficient%**
Normal control	4.57±0.38	0.26±0.10	0.16±0.05
Cy(100 mg/kg)	4.80±0.45	0.21±0.06^#^	0.04±0.01^##^
ZYQL-A(100 mg/kg)	4.32±0.63	0.29±0.10	0.17±0.05
ZYQL-B(200 mg/kg)	4.39±0.25	0.31±0.06	0.15±0.03
ZYQL-C(400 mg/kg)	4.41±0.48	0.27±0.07	0.15±0.01
ZYQL-A(100 mg/kg)+Cy	4.02±0.21	0.25±0.05	0.12±0.02^**^
ZYQL-B(200 mg/kg)+Cy	4.28±0.56	0.27±0.03^*^	0.13±0.03^**^
ZYQL-C(400 mg/kg)+Cy	4.38±0.40	0.27±0.04^*^	0.13±0.04^**^

Data is expressed as the mean±SD (n = 10).

^##^ Significant difference at *p*<0.01 levels compared with normal control group.

^#^ Significant difference at *p*<0.05 levels compared with normal control group.

^**^ Significant difference at *p*<0.01 levels compared with Cy-treated group.

^*^ Significant difference at *p*<0.05 levels compared with Cy-treated group.

### Effect of ZYQL on phagocytotic function of mononuclear phagocytic system

In Table 2, the carbon clearance index of Cy-intoxicated group was significantly decreased compared with the normal control group (*p*<0.01, Table 2). And in the immunosuppressed mice group, the carbon clearance were significantly enhanced after treated with ZYQL (100, 200 and 400 mg/kg) for fourteen days, which compared with Cy-intoxicated group (*p*<0.05-0.01, Table 2). On the contrary, there was no enhancement in the phagocytotic function in the normal mice treat with 100 and 200 mg/kg ZYQL compared with the normal control group. Nevertheless, treated with 400 mg/kg ZYQL alone could increase the phagocytotic function compared with the normal control group (*p*<0.05, Table 2).

**Table 2:** Effect of ZYQL on phagocytotic function of mononuclear phagocytic system

**Treatment**	**Liver+spleen**^**a**^	**A**^**b**^
Normal control	1.65±0.15	3.87±0.74
Cy(100 mg/kg)	1.18±0.14^##^	3.04±0.65^##^
ZYQL-A(100 mg/kg)	1.70±0.13	3.99±0.91
ZYQL-B(200 mg/kg)	1.80±0.16	4.23±0.98
ZYQL-C(400 mg/kg)	1.85±0.21	4.35±0.87^#^
ZYQL-A(100 mg/kg)+Cy	1.43±0.15	3.69±0.46^*^
ZYQL-B(200 mg/kg)+Cy	1.64±0.18^*^	3.97±0.58^*^
ZYQL-C(400 mg/kg)+Cy	1.72±0.16^*^	4.11±0.62^**^

Data is expressed as the mean±SD (n = 10).

^a^ g.

^b^ Carbon clearance index.

^##^ Significant difference at *p*<0.01 levels compared with normal control group.

^#^ Significant difference at *p*<0.05 levels compared with normal control group.

^**^ Significant difference at *p*<0.01 levels compared with Cy-treated group.

^*^ Significant difference at *p*<0.05 levels compared with Cy-treated group.

### Effect of ZYQL on cytokine secretion levels and lysozyme activity in normal and immunosuppressed mice

In Table 3, the levels of IL-6, IFN-γ and LSZ were significantly decreased in the Cy-intoxicated group compared with the normal control group (*p*<0.01, Table 3). Compared with the Cy-intoxicated group, ZYQL enhanced the production of both IL-6 and IFN-γ in the serum of immunosuppressed mice (*p*<0.01, Table 3). While, there is no significant difference compared with normal mice. Furthermore, ZYQL (200 and 400 mg/kg) significantly increased the activity of LSZ in normal mice compared with the normal control group (*p*<0.05-0.01, Table 3), and similar in case of the immunosuppressed mice treated with 100, 200 or 400 mg/kg of ZYQL, the activity of LSZ was significantly increased compared with Cy only treated group (*p*<0.05-0.01, Table 3).

**Table 3:** Effect of ZYQL on cytokine secretion levels and LSZ in normal and immunosuppressed mice

**Treatment**^∆^	**IL-6**^**a**^	**IFN-γ**^**b**^	**LSZ**^**c**^
Normal control	74.47±15.12	246.44±32.62	6.31±0.98
Cy(100 mg/kg)	40.56±9.76^##^	80.96±10.83^##^	3.87±0.67^##^
ZYQL-A(100 mg/kg)	81.60±24.82	262.52±55.74	7.03±1.05
ZYQL-B(200 mg/kg)	89.04±15.57	277.61±68.42	7.67±1.12^##^
ZYQL-C(400 mg/kg)	86.98±11.97	256.81±46.83	7.30±0.91^#^
ZYQL-A(100 mg/kg)+Cy	80.54±11.37^**^	185.34±28.81^**^	4.57±0.85^*^
ZYQL-B(200 mg/kg)+Cy	101.74±30.72^**^	231.84±58.32^**^	8.87±0.76^**^
ZYQL-C(400 mg/kg)+Cy	79.24±16.66^**^	230.16±31.63^**^	7.98±0.98^**^

Data is expressed as the mean±SD (n = 10).

^a^ pg/mL.

^b^ pg/mL.

^c^ μg/mL.

^##^ Significant difference at *p*<0.01 levels compared with normal control group.

^#^ Significant difference at *p*<0.05 levels compared with normal control group.

^**^ Significant difference at *p*<0.01 levels compared with Cy-treated group.

^*^ Significant difference at *p*<0.05 levels compared with Cy-treated group.

^∆^ Significant difference at *p*<0.05 (f_IFN-γ_ = 3.692, f_LSZ_ = 3.845) levels compared between cyclophosphamide- untreated groups and cyclophosphamide-treated groups (two-way ANOVA followed by LSD’s test).

### Effect of ZYQL on spleen antioxidant enzyme activities in normal and immunosuppressed mice

Since oxidative stress contributes to the development of Cy-induced immunosuppression [24], the levels of spleen antioxidant enzymes SOD, CAT and GSH-Px were measured. Amounts of SOD, CAT and GSH-Px were significantly diminished in the Cy-intoxicated group compared with the normal control (*p*<0.01, Table 4). Pretreatment with 100, 200 and 400 mg/kg of ZYQL significantly raised the antioxidant enzyme levels as compared with the Cy-intoxicated group in the immuonsuppressed mice (*p*<0.05-0.01, Table 4), and similar with the normal mice treated with 200 or 400 mg/kg of ZYQL compared with normal control group (*p*<0.01, Table 4).

**Table 4:** Effect of ZYQL on spleen antioxidant enzyme activities in normal and immunosuppressd mice

**Treatment**^∆^	**GSH-Px**^**a**^	**CAT**^**b**^	**SOD**^**c**^
Normal control	1029.23±79.08	30.28±2.91	103.07±5.52
Cy(100 mg/kg)	566.53±26.56^##^	17.23±1.48^##^	54.66±9.61^##^
ZYQL-A(100 mg/kg)	1097.86±96.38	33.87±1.05	106.14±14.07
ZYQL-B(200 mg/kg)	1181.81±44.51^##^	45.02±6.00^##^	131.75±37.18^##^
ZYQL-C(400 mg/kg)	1167.57±39.34^##^	42.96±6.94^##^	140.00±20.25^##^
ZYQL-A(100 mg/kg)+Cy	646.71±42.88^*^	19.85±1.65^**^	79.16±12.69^**^
ZYQL-B(200 mg/kg)+Cy	724.57±56.51^**^	23.57±0.66^**^	87.46±9.54^**^
ZYQL-C(400 mg/kg)+Cy	722.08±112.92^**^	20.24±1.30^**^	81.66±10.26^**^

Data is expressed as the mean±SD (n = 10).

^a^ μmol/g protein.

^b^ U/mg protein.

^c^ U/mg protein.

^##^ Significant difference at *p*<0.01 levels compared with normal control group.

^**^ Significant difference at *p*<0.01 levels compared with Cy-treated group.

^*^ Significant difference at *p*<0.05 levels compared with Cy-treated group.

^∆^ Significant difference at *p*<0.05 (f_CAT_ = 9.476, f_SOD_ = 2.613) levels compared between cyclophosphamide-untreated groups and cyclophosphamide-treated groups (two-way ANOVA followed by LSD’s test).

## Discussion

In the present investigation, we examined the protective effects of ZYQL on the immune function of normal and immunosuppressed mice in vivo, which was identified as an health food for a thousands of years.

Non-specific immunity is an important part in the immunity system of the body. Synchronously, it is well known that macrophages play a key role in the host defense mechanism and many immunoregulants activate immune responses primarily by activation of macrophages, although direct activation of B cells and other immune cells also are implicated [25]. So, the phagocytotic function of mononuclear phagocytic system is regarded as the marker to measurement the non-specific immunity [26]. And the macrophage phagocytotic index could be reflect the phagocytotic function. In this study, the phagocytotic function of mononuclear phagocytic system was detected with carbon clear up methods, which displayed that ZYQL could increase the carbon clearance index in the immunosuppressed mice, and similarly, 400 mg/kg ZYQL exhibited the enhancement of the carbon clearance index in the normal mice. This result suggest that ZYQL may enhance the non-specific immunity by increasing the phagocytic activity of macrophages.

LSZ is a small, basic, globular mucopolysaccharide protein consisting of a chain with 129 amino acids cross-linked by four or five disulphate bridges, which move actively through the cytoplasm to carry out their intracellular digestion (i.e., in heterophagy and autophagy). The present work exhibit that ZYQL (100, 200 and 400 mg/kg) treated groups significantly improved the serum LSZ activity in immunosuppressed mice (*p*<0.05-0.01), and ZYQL (200 and 400 mg/kg) treated groups significantly improved LSZ activity (*p*<0.05-0.01) in normal mice. It was shown that LSZ has many functions such as phagocytosis, anti-bacterium, anti-virus and anti-tumour, and plays an important role in the clearance of exogenous foreign bodies and endogenous residues.

Furthermore, cell-mediated immune defense played a key role in anti-tumor activity and was mediated specifically by T cells and nonspecifically by macrophages and NK cells. IFN-γ is a potent immunomodulatory cytokine, it is a member of the interferon family that regulates anti-proliferative, anti-angiogenic, anti-cancer effects, as well as, adaptive immune responses [27]. IL-6 is a multifunctional cytokine, which is produced by both lymphoid and non-lymphoid cells including macrophages, fibroblasts and endothelial cells and involved, in antigen-specific immune responses and inflammatory reactions [28]. In the present study, we demonstrated that ZYQL strongly increased the levels of IFN-γ and IL-6 in the immunosuppressed mice, while it did not significantly change these cytokines in normal mice.

In addition, SOD has been reported as one of the most important enzymes in the enzymatic antioxidant defense system. It scavenges the superoxide anion to form hydrogen peroxide and thus diminishing the toxic effect caused by this radical. ZYQL causes a significant increase in spleen SOD activity in both normal and immunosuppressed mice (*p*<0.01), except in the group of 100 mg/kg ZYQL, and thus reduces reactive free radical induced oxidative damage to spleen.

CAT is an enzymatic antioxidant widely distributed in all animal tissues, and the highest activity is found in the red cells. CAT decomposes hydrogen peroxide and protects the tissues from highly reactive hydroxyl radicals [29]. Therefore, reduction in the activity of CAT may result in a number of deleterious effects due to the assimilation of superoxide radical and hydrogen peroxide. All doses of ZYQL (100, 200 and 400 mg/kg) are significantly increased the level of CAT both in normal and immunosuppressed mice (*p*<0.01), except in the group treated with 100 mg/kg ZYQL only.

GSH-Px is the substrate of glutathione, which could removes free radical species such as hydrogen peroxide, superoxide radicals and maintains membrane protein thiols [30]. And it combined SOD and CAT to formed an antioxidant defense system of the body. Decreased the level of GSH-Px exhibits in Cy-treated mice compared the normal control group (*p*<0.01). Administration of ZYQL (100, 200 and 400 mg/kg) significantly increased the level of GSH-Px in normal and immunosuppressed mice (*p*<0.05-0.01), except in the group treated with 100 mg/kg ZYQL only.

Moreover, thymus and spleen are the important immune organs of the animal. And their weight can relatively reflect the immune function. Thus, spleen index and thymus index are considered as the most elementary and conventional index, which have been generally used to evaluate the whole immune state of the organism. In the present work, ZYQL (200 and 400 mg/kg) significantly increased thymus index and spleen index in immunosuppressed mice (*p*<0.05 and *p*<0.01, respectively) compare with the Cy-intoxicated group, while there is no significant difference in thymus and spleen indices in the normal mice treated with ZYQL compare with the normal control group. This also indicated that ZYQL is helpful for the immunosuppressed mice.

## Conclusions

In conclusion, the present reasearch demonstrated that ZYQL has the beneficial protective effects on the immune function of normal and immunosuppressed mice in vivo. Although the exact underlying mechanism of ZYQL is yet unknown, based on the results presented above, we can conclude that ZYQL has the protective effects on the immune function by regulating cytokines expression, which raised the levels of IL-6 and IFN-γ in serum. Moreover, the carbon clearance experiment and the lysozyme activity also suggests that macrophages involved in non-specific immunity were primary activated, and helper T cell were secondarily affected by ZYQL. On the other hand, the study showed that ZYQL had the antioxidant effects in immune system by enhancing the antioxidant enzyme activity of SOD, CAT and GSH-Px in vivo. In addition, ZYQL could also increase the thymus index and the spleen index in the immunosuppressed mice. All the above was suggested that ZYQL could enhance the immunological activity of the Cy-induced immunosuppressed mice.

## Competing interests

The authors declare that they have no competing interests.

## Authors’ contributions

The author, HYG, HYW, FZS carried out the whole experiment. YH, XWD, LJA prepared the experimental drug. HYG and JHW participated in the design of the study, performed the statistical analysis and drafted the manuscript. All authors read and approved the final manuscript.

## Pre-publication history

The pre-publication history for this paper can be accessed here:

http://www.biomedcentral.com/1472-6882/13/252/prepub
